# Social and environmental factors affect tuberculosis related mortality in wild meerkats

**DOI:** 10.1111/1365-2656.12649

**Published:** 2017-04-03

**Authors:** Stuart Patterson, Julian A. Drewe, Dirk U. Pfeiffer, Tim H. Clutton‐Brock

**Affiliations:** ^1^ Veterinary Epidemiology, Economics and Public Health GroupRoyal Veterinary CollegeUniversity of LondonHawkshead LaneHatfield AL9 7TAUK; ^2^ School of Veterinary MedicineCity University of Hong KongTat Chee AvenueKowloon, Hong Kong SAR; ^3^ Large Animal Research GroupDepartment of ZoologyUniversity of CambridgeDowning StreetCambridge CB2 3EJUK; ^4^ Mammal Research InstituteUniversity of PretoriaHatfield, PretoriaSouth Africa

**Keywords:** heterogeneity, *Mycobacterium suricattae*, targeted control, wildlife disease

## Abstract

1. Tuberculosis (TB) is an important and widespread disease of wildlife, livestock and humans world‐wide, but long‐term empirical datasets describing this condition are rare. A population of meerkats (*Suricata suricatta*) in South Africa's Kalahari Desert have been diagnosed with *Mycobacterium suricattae*, a novel strain of TB, causing fatal disease in this group‐living species.
2. This study aimed to find characteristics associated with clinical TB in meerkats. These characteristics could subsequently be used to identify ‘at‐risk’ animals within a population, and target these individuals for control measures.
3. We conducted a retrospective study based on a unique, long‐term life‐history dataset of over 2000 individually identified animals covering a 14‐year period after the first confirmatory diagnosis of TB in this population in 2001. Individual‐ and group‐level risk factors were analysed using time‐dependent Cox regression to examine their potential influence on the time to development of end‐stage TB.
4. Cases of disease involved 144 individuals in 27 of 73 social groups, across 12 of 14 years (an incidence rate of 3·78 cases/100 study years). At the individual level, increasing age had the greatest effect on risk of disease with a hazard ratio of 4·70 (95% CI: 1·92–11·53, *P* < 0·01) for meerkats aged 24–48 months, and a hazard ratio of 9·36 (3·34–26·25, *P* < 0·001) for animals aged over 48 months (both age categories compared with animals aged below 24 months). Previous group history of TB increased the hazard by a factor of 4·29 (2·00–9·17, *P* < 0·01), and an interaction was found between this variable and age. At a group level, immigrations of new group members in the previous year increased hazard by a factor of 3·00 (1·23–7·34, *P* = 0·016). There was weaker evidence of an environmental effect with a hazard ratio for a low rainfall (<200 mm) year of 2·28 (0·91–5·72, *P* = 0·079).
5. Our findings identify potential individual characteristics on which to base targeted control measures such as vaccination. Additional data on the dynamics of the infection status of individuals and how this changes over time would complement these findings by enhancing understanding of disease progression and transmission, and thus the implications of potential management measures.

## Introduction

Heterogeneity in disease susceptibility influences transmission routes and frequencies within a population (Dwyer, Elkinton & Buonaccorsi 1997; Barlow 2000; McCallum, Barlow & Hone 2001). An understanding of the magnitude and distribution of these differences represents an important component of our ability to predict the dynamics of disease, and so may allow us to intervene and reduce infection transmission. This study asks whether factors such as sex, age and social characteristics influence the likelihood that an individual will develop the clinical disease tuberculosis (TB) in a population of meerkats (*Suricata suricatta*).

Tuberculosis is a chronic disease of humans, livestock and wildlife which has public health, economic and conservation importance (Gortazar & Cowan 2013). Caused by members of the Mycobacterium Tuberculosis Complex (MTC), it is known to affect a wide range of wild mammal species including buffalo (*Syncerus caffer*), lion (*Panthera leo*) and wild boar (*Sus scrofa*) (De Garine‐Wichatitsky *et al*. 2013). Evidence from badgers (*Meles meles*) suggests that individuals become increasingly infectious as disease progresses (Gallagher 1998; Corner 2006) and the extent to which lesions shed bacteria varies with disease stage (Gavier‐Widen *et al*. 2001). Individuals with clinical disease are therefore likely to be the most infectious animals in the population, and their characteristics carry important epidemiological information as to where transmission is likely to be occurring.

Tuberculosis was first diagnosed in a long‐term study population of meerkats in the Northern Cape of South Africa in 2001 (Drewe *et al*. 2009a), and cases have been recorded in 12 of the following 14 years. The social behaviours of meerkats are implicated in spread of infection within their population (Drewe *et al*. 2011). In each meerkat group, a dominant female and a dominant male produce over 80% of offspring reared (Clutton‐Brock *et al*. 1999) with pup care being shared by all group members (Doolan & Macdonald 1997; Clutton‐Brock *et al*. 2001). Our study uses long‐term data collected from a wild population of meerkats in the southern Kalahari (Clutton‐Brock *et al*. 1998). The animals live freely in stable, mixed sex, hierarchical groups of up to 40 individuals (Hodge *et al*. 2008) with typically 15–20 groups being observed at any one time period. Individuals were uniquely identified, through both subcutaneous microchips, and the regular application of patterns of hair dye, and their birth dates were known. Meerkats were habituated to human contact, allowing for close observation and collection of data on their behaviours, life history and changes in bodyweight.

A novel member of the MTC, *Mycobacterium suricattae*, has been identified in meerkats showing clinical signs of TB (Parsons *et al*. 2013). The pathology of TB in meerkats has been described (Drewe *et al*. 2009a), with grossly enlarged lymph nodes (chiefly the submandibular and medial retropharyngeal) being characteristic of the disease. Drewe *et al*. (2009a) found evidence of the presence of mycobacteria either by histology or culture in 100% of 52 meerkats showing submandibular swelling, suggesting this sign is pathognomonic for TB in this species. Transmission is believed to occur by bite wounds and the respiratory route, with aggressive behaviours, and allo‐grooming identified as particularly high‐risk activities (Drewe 2010). Aggressive interactions between individuals maintain the dominance hierarchy (Kutsukake & Clutton‐Brock 2006a), with submissive behaviours such as grooming reinforcing social position (Kutsukake & Clutton‐Brock 2006b; Madden *et al*. 2011). Interactions between individuals in different groups tend to be aggressive, either involving roving males, or whole group encounters (Young, Spong & Clutton‐Brock 2007; Drewe, Madden & Pearce 2009b).

A novel member of the MTC, *Mycobacterium suricattae*, has been identified in meerkats showing clinical signs of TB (Parsons *et al*. 2013). The pathology of TB in meerkats has been described (Drewe *et al*. 2009a), with grossly enlarged lymph nodes (chiefly the submandibular and medial retropharyngeal) being characteristic of the disease. Drewe *et al*. (2009a) found evidence of the presence of mycobacteria either by histology or culture in 100% of 52 meerkats showing submandibular swelling, suggesting this sign is pathognomonic for TB in this species. Transmission is believed to occur by bite wounds and the respiratory route, with aggressive behaviours, and allo‐grooming identified as particularly high‐risk activities (Drewe 2010). Aggressive interactions between individuals maintain the dominance hierarchy (Kutsukake & Clutton‐Brock 2006a), with submissive behaviours such as grooming reinforcing social position (Kutsukake & Clutton‐Brock 2006b; Madden *et al*. 2011). Interactions between individuals in different groups tend to be aggressive, either involving roving males, or whole group encounters (Young, Spong & Clutton‐Brock 2007; Drewe, Madden & Pearce 2009b).

Haematogenous spread leads to disseminated infection, with the spleen, liver, lung and head lymph nodes the most common sites of detection (Drewe *et al*. 2009a). Management policy at the study site is to euthanase meerkats at the point at which a swollen lymph node bursts. This is in an attempt to limit onward transmission to other meerkats being studied as habituation of individuals is costly in terms of time. Prior to the implementation of this policy in the 1990s, diseased individuals within the population all died naturally within less than 6 months of the observation of a lymph node swelling. TB is believed to be endemic in the population due to the regularity and frequency of disease incidence.

In this paper, we identify factors that affect the risk that wild meerkats will develop clinical signs of TB. We initially look at the distribution of cases and the incidence within the population. We then ask whether factors such as age, sex and dominance status affect the length of time an individual takes to become a case. Finally, we analyse the factors that were associated with an infected group developing its first case of disease. The results are likely to aid understanding of the epidemiology of TB in this, and potentially other, social living wild animal host species, as well as informing the management of this difficult disease.

## Materials and methods

### Ethics statement

Data collection at the Kuruman River Reserve was carried out under ethics approval from the University of Pretoria's ethics committee, and with the permission of the Northern Cape Department of Environment and Nature Conservation. The study design was additionally approved by the Royal Veterinary College's research committee (authorisation PPH_01355).

### Study population

All data were collected from a free‐ranging, naturally regulated population of approximately 250 (at any one time) free‐living meerkats at the Kuruman River Reserve (26°58′S, 21°49′E) in the Northern Cape of South Africa. Animals that were alive and recorded in the Kalahari Meerkat Project's long‐term dataset at any point between 1 January 2002 and 30 June 2015 (the study cut‐off point) were included in the analysis totalling over 2000 individuals. Meerkats were free to enter or leave the studied social groups throughout the study period, and new social groups were formed from group splits and recruitment of new wild groups while others moved out of the area or died out.

### Data collection

Habituated groups were visited at least once every week during the study period, and each meerkat was visually checked for signs of disease from a distance of less than a metre. All life‐history data were collected according to previously described project protocols (Clutton‐Brock *et al*. 1999). Dominance was determined through observation of submission behaviours and dominance assertions during routine data collection sessions, a dominant male and dominant female being identified in each group (Young, Spong & Clutton‐Brock 2007; Spong *et al*. 2008; Kutsukake & Clutton‐Brock 2010). Group compositions were recorded every 2–3 days (Mares *et al*. 2014), and information was extracted for occurrence of immigrations and numbers of adult subordinate males within a group. The latter number was used as a proxy for the magnitude of roving behaviour (temporary inter‐group movements of males) as these animals are the most likely individuals to engage in prospecting behaviour for mating opportunities (Mares *et al*. 2014). Data were recorded in a customised Microsoft Access database. A record was kept detailing all signs of overt illness observed in individual meerkats throughout the study period. This included information on which animals were showing visual signs of TB, the most common of which was visibly enlarged submandibular and medial retropharyngeal lymph nodes. Animals with pronounced submandibular swellings were euthanased using an overdose of intravenous sodium pentobarbitone injected under gaseous anaesthesia, at the point at which the enlarged lymph nodes burst. Rainfall data were collected using a permanent onsite rain gauge (Bell *et al*. 2014; Huchard *et al*. 2014), with additional meteorological information obtained from NASA's GES DISC (Goddard Earth Sciences Data and Information Services Center) Giovanni online data system (Acker & Leptoukh 2007). Data were extracted and manipulated in Microsoft Excel to create records for each individual in the study. The resulting dataset was imported into ‘R’ version 3.2.3 (R Core Team, 2013) for statistical analysis.

### Study design

This was a retrospective study of pre‐existing records. The case definition was that an individual must have been euthanased due to advanced signs of TB, and there must have been a record of a persistently enlarged submandibular lymph node prior to euthanasia. Animals were assigned a binary TB status with 1 being a case. Individuals entered the study at birth, and the final dates were either the point at which the animal left the study population (either through loss to follow‐up, euthanasia or another known cause of death) or the study end point (30 June 2015). A binary variable was created, ‘Previous TB in group’ and an individual was given a status of 1 if it was alive and a member of a social group at any point after the first TB euthanasia occurred in that group. Individuals’ records were broken down into 3‐month periods to allow the explanatory variables to change over time. The year and the individual's age were calculated on the first day of each time period.

In analysis of the effects of group characteristics on the risk of visual signs of disease, groups were either recorded as showing a previous history of TB (if any TB‐related death was ever known to have occurred within the group), or as having no previous history. For both levels of analysis, time was expressed in days, reflecting the regularity at which the animals were observed.

### Data analysis

Univariable analysis was initially performed on all recorded explanatory variables using time‐dependent Cox regression (Cox & Oakes 1984; Van Dijk *et al*. 2008) for both the individual animal and the group analyses using the Survival package in R (Therneau & Grambsch 2000).

For the individual animal analysis, the Cox model utilised data on the time until an individual became a case, while being able to take account of left and right censored information, and the influence that the explanatory variables had on the probability of survival beyond a particular time. Similarly, for the group‐level analysis, the model used the time until the first individual case was observed within the group. Subsequently, a multivariable analysis was carried out including all terms for which *P* < 0·2 in the univariable analysis, in a forward‐stepwise process and testing the significance of changes to the model using analysis of deviance for a Cox model (Therneau & Grambsch 2000). Individuals are free to move between social groups, and so were not necessarily born in the group at which they were being observed. In order to account for the potential influence of natal group, a gamma frailty term was included for this random effect (Hougaard 1995). Explanatory variables included for individuals were age, year of birth, previous cases of TB within the social group, sex and dominance status. Calendar year was categorised into three time periods (2002–2005, 2006–2010 and 2011–2015) to maximise sample size and age was expressed in whole years. Group size was grouped (<10, 11–20, 21–30 and >30) based on the distribution of the data, and rainfall was expressed as above or below the median level (200 mm). For the group‐level analysis, the variables included were the average group size, the year (grouped as for the individuals), number of adult subordinate males in the group (categorised as 0–4, 5–9 and >9), rainfall, presence of dominance changes and occurrence of immigration events. Dominance change refers to whether the group experienced a change in the dominant male and/or female during that time period, while ‘6‐month’ and ‘12‐month’ dominant changes refer to this occurring in the preceding 6 or 12 months respectively. Similarly, immigration refers to whether animals immigrated into that group during the described time periods. Eligible rovers were defined as subordinate males aged over 1 year, and the number given for the previous 12 months is the highest number eligible at any point during this time. Possible first‐order interactions between these variables were explored and included where appropriate, and the validity of the proportional hazards assumption was tested using Schoenfeld residuals (Grambsch & Therneau 1994).

## Results

**Figure 1:**
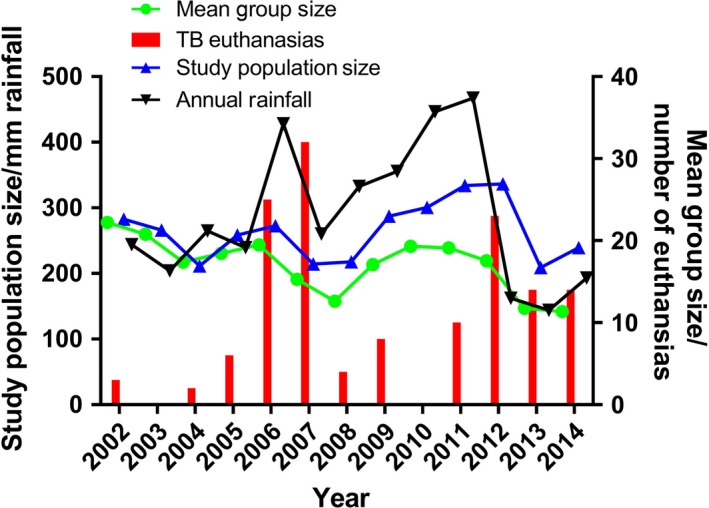
Annual meerkat tuberculosis (TB) case rate at the study site. Total annual rainfall per calendar year (2002–2014) at the Kalahari Meerkat Project is displayed alongside the mean group size, the total number of meerkats in the study population that year and the number of euthanasias performed in animals meeting the criteria for advanced TB disease.

**Figure 2:**
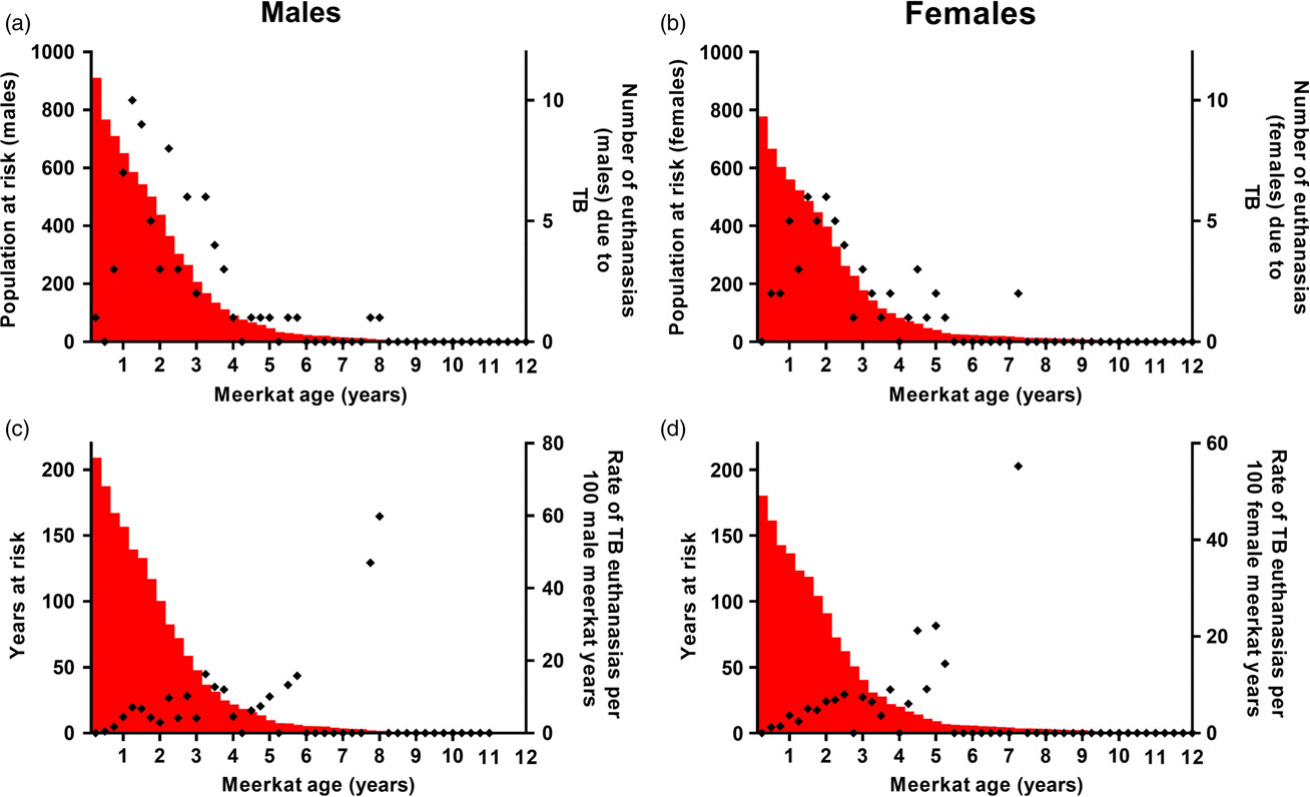
The relationship between meerkat age and risk of euthanasia due to tuberculosis (TB). (a) and (b) show the total number of males and females that reached each age category, the population at risk, in red, and the points show the number of animals euthanased at each age. (c) and (d) show the total number of animals that contributed to each age category in red, for both males and females, and the points indicate the incidence of TB euthanasias in each category. While the number of TB‐related deaths decreases with age, the incidence rate can be seen to increase.

A total of 2388 meerkats were followed during the 13·5‐year study period, each individual making a median contribution of 455 days (range: <1 day to 4468 days). These individuals belonged to 73 different social groups, and 307 meerkats had been recorded as dominant animals at some point (median dominance duration 293 days, range: 1–3835 days). The median number of animals in each group over the total time period was 15 (range: 1–46). The sex of 2091 (87·6%) of the 2388 study animals was known; of these, 1165 (55·7%) were male. In total, 144 individuals (6·03% of all animals studied) met the case definition for TB, giving an incidence rate of 3·78 cases per 100 animal‐years studied. These TB cases were found in 27 social groups (37·0% of all groups followed), during 12 of the 14 study years (see Fig. 1). Individuals aged under 2 years contributed 2536 years (70·6%) to the total analysis period (see Fig. 2). Figures 2(a) and (b) show that the greatest number of TB‐related deaths occurred in animals aged 1–3 years, while Figs 2(c) and (d) show that the risk of euthanasia actually continues to increase beyond this age but is masked by a smaller population at risk.

**Figure 3:**
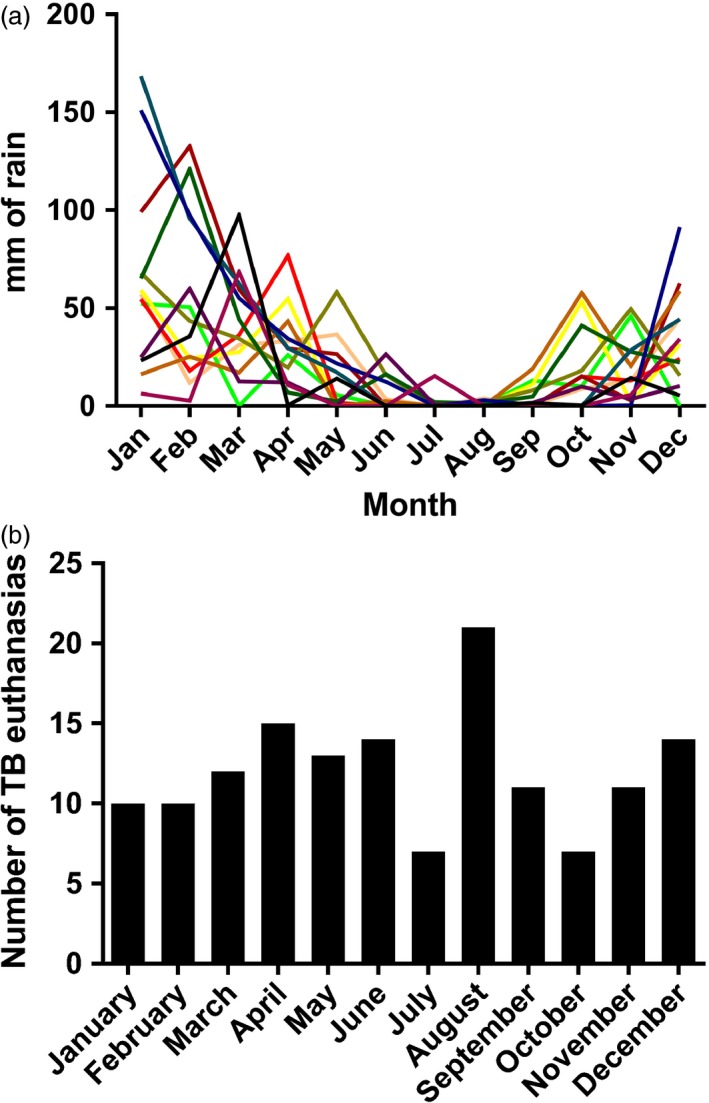
Annual and monthly rainfall. Monthly rainfall and euthanasia figures at the study site using data from 2002 to 2014. (a) Monthly rainfall figures for each year across the study period showing a consistent annual pattern; each year is represented by a different coloured line; (b) The number of meerkats euthanased due to tuberculosis by month, showing no pattern throughout the year.

Median annual rainfall was 262 mm (range 115–473 mm), with annual peaks in 2006 and 2011 (Fig. 3). Despite a clear pattern in the environmental conditions across the year, there is no reflection of this in the incidence of euthanasia (Fig. 3). Numbers of non‐habituated groups in the area were unknown, and in the absence of this information, changes in mean group size were adopted as a proxy for changes in the true, local population size. Despite peaking during times of highest rainfall, both the total study population and the mean size of each study group dropped following years with peak rainfall. The highest numbers of meerkats being euthanased due to TB occurred in 2007 and 2012. Monthly rainfall varied at the study site throughout the year, with June, July and August being the driest months in all years. Despite this clear seasonal variation in rainfall, no relationship between monthly rainfall and incidence of euthanasias due to TB was evident (*P* = 0·5).

Despite there being no sex bias in individual risk of TB, when the first case in each group was examined, males were found to be disproportionally represented; the index case was male in 18 (75%) of the groups that became diseased. A distinct individual first case was noted in 24 groups, while the remainder had two or more contemporaneous cases. In 13 cases (54%), the index was a subordinate adult male, a dominant male on four occasions (17%) and a sub‐adult male in a single case (4%). Dominant females were the first case on four occasions (17%) and subordinate adult females in two cases (8%).

### Univariable analysis

At the individual animal level, effects on the hazard of euthanasia associated with TB cases were found for age (*P* < 0·001) and a prior case in the social group (*P* < 0·01) when considering 1901 animals of known sex and birthdate (Table 1). Calendar year (*P* = 0·09) was also included in the subsequent multivariable analysis.

**Table 1:** Univariable analysis of individual‐ and group‐level risk factors. Results of a univariable analysis of data collected from 1901 individuals in 73 social groups at the Kalahari Meerkat Project from 2002 to 2015, using time‐dependent Cox regression. Hazards were calculated for the likelihood of an individual meerkat being euthanased due to advanced tuberculosis (TB), and for an uninfected group to show its first case, using 3‐month time intervals

Variable	Hazard ratio	95% Confidence interval	Wald test *P* value
Individual risk			
Year			
2002–2005 baseline			0·09
2006–2010	3·30	0·52–20·82	
2011–2015	0·55	0·04–8·14	
Sex			
Female			0·35
Male	1·18	0·84–1·66	
Previous group TB			
No			<0·0001
Yes	3·98	2·60–6·10	
Dominant status			
Never			0·42
Ever	0·86	0·59–1·25	
Age (years)			
<1			<0·0001
1	3·30	1·92–5·68	
2	3·73	2·09–6·66	
3	4·75	2·50–9·04	
4	5·66	2·61–12·27	
5	3·72	1·09–12·62	
6+	2·66	0·91–7·84	
Group risk			
Dominance change			
No			0·86
Yes	1·09	0·40–2·98	
6‐month dominance change			
No			0·42
Yes	0·68	0·26–1·74	
12‐month dominance change			
No			0·27
Yes	0·62	0·27–1·45	
Group size			
<10			0·66
11–20	1·25	0·45–3·46	
21–30	0·91	0·24–3·49	
>31	0·45	0·05–3·60	
Rainfall			
<200 mm			0·05
>200 mm	0·39	0·15–1·00	
Immigration			
No			0·19
Yes	2·09	0·70–6·26	
6‐month immigration			
No			0·11
Yes	2·23	0·84–5·96	
12‐month immigration			
No			0·01
Yes	3·13	1·29–7·58	
Number of eligible rovers			
0–4			0·83
5–9	1·03	0·41–2·59	
10+	0·58	0·15–2·18	
Number of eligible rovers in the preceding 12 months			
0–4			0·79
5–9	1·42	0·52–3·88	
10+	1·15	0·39–3·41	
Year			
2002–2005			0·13
2006–2010	1·32	0·35–4·93	
2011–2015	2·69	0·82–8·82	

At the group level, there was strong evidence that one or more immigrations into the group within the previous 12 months (*P* = 0·01) and low rainfall (<200 mm per year, *P* = 0·05) increased the hazard ratio for a group becoming diseased. Along with these variables, Year (*P* = 0·13) was carried forward to the multivariable model.

### Multivariable analysis

The multivariable model (Table 2) revealed a hazard ratio of TB in 2006–2010 of 2·86 (95% CI: 0·47–17·54, *P* < 0·001), and a hazard ratio of 0·38 (0·02–6·80, *P* < 0·001) in 2011–2015, compared with 2002–2005. The variance for the frailty term (natal group) was 1·11 (*P* < 0·001). A history of TB in the group increased the hazard by a factor of 4·29 (2·00–9·17, *P* < 0·01). Compared to the youngest individuals, age increased the hazard by a factor of 4·70 (1·92–11·53, *P* < 0·01) for 2‐ to 4‐year olds, and 9·36 (3·34–26·25, *P* < 0·001) for the over 4‐year olds. There was an interaction between previous TB history and age (*P* < 0·0001).

**Table 2:** Multivariable analysis of individual‐ and group‐level risk factors. Results of a multivariable analysis of data collected from 1901 individuals in 73 social groups at the Kalahari Meerkat Project between 2002 and 2015, using time‐dependent Cox regression. Hazards were calculated for the likelihood of an individual meerkat being euthanased due to advanced tuberculosis (TB), and for an uninfected group to show its first case, using 3‐month time intervals

Variable	Hazard ratio	95% Confidence interval	*P* value
Individual risk			
Year			
2002–2005 baseline			
2006–2010	2·86	0·47–17·54	<0·001
2011–2015	0·38	0·02–6·80	<0·001
Previous group TB			
No			
Yes	4·29	2·00–9·17	<0·01
Age (years)			
<2			
2–4	4·70	1·92–11·53	<0·01
4+	9·36	3·34–26·25	<0·001
Variance of frailty term for Natal group = 1·111, *P* < 0·001			
Group risk			
12‐month immigration			
No			0·016
Yes	3·00	1·23–7·34	
Rainfall			
<200 mm			0·079
>200 mm	0·44	0·17–1·1	

At the group level, there was strong evidence that immigration into the group in the previous 12 months increased the hazard by a factor of 3·00 (1·23–7·34, *P* = 0·016), and some evidence that more than 200 mm rainfall over that year was protective, with a hazard ratio of 0·44 (0·17–1·10, *P* = 0·079). The proportional hazards assumption was met, and no interactions were observed in this analysis.

## Discussion

Around 6% of individuals in our study population are known to have died as a result of TB. The age of an individual and the individual's social group's TB history were the main factors affecting the risk that individual meerkats in our study population would die from TB. There was little evidence of any consistent sex differences in risk, although males were more often the first animal in a group to display visual signs of TB. Research in other species such as badgers and deer suggests that these individuals showing advanced disease are the most likely to become increasingly infectious (Gallagher 1998; Lugton *et al*. 1998; Gallagher & Clifton‐Hadley 2000; Corner, Murphy & Gormley 2011; Delahay *et al*. 2013). Identifying these individuals may offer the opportunity to target individuals for disease control measures (e.g. vaccination or culling) before they become infectious, and so reduce disease transmission. A better understanding of which animals are most likely to show advanced stages of disease will also improve our knowledge of the important role of individuals within the population's transmission dynamics. It is possible that these transmission routes also include other species in the same environment, although no studies have been done to investigate this.

Despite suggestions that in female‐dominated social systems, such as in meerkats, testosterone‐linked immunosuppression may lead to a predominance of females among diseased animals (Smyth & Drea 2015), there is no evidence found here for sex bias in rates of *disease*. Similar findings are reported in African buffalo with TB (Renwick, White & Bengis 2007), despite males, through physiological and behavioural differences, often having a greater predilection for contracting infections (Cross *et al*. 2009). The outcome of interest in the present study was disease rather than infection, and the possibility exists that males could have higher infection rates than females, but with infected females progressing to clinical disease due to sex‐linked immunosuppression. Female meerkats have previously been shown to have increased androgen levels, linked to an immunocompromised state (Smyth *et al*. 2016). Alternatively, infected males may be more likely to be lost to follow‐up before they develop signs. Both Wilkinson *et al*. (2000) and Tomlinson *et al*. (2013b) found evidence that infected female badgers have longer survival times than males, and Jackson (1995) reported a greater infection risk in male possums (*Trichosurus vulpecula*) with TB.

In meerkats, sexually mature males periodically leave their social group to rove for potential mates at other groups, and this behaviour may play an important role in introducing infection to a social group (Drewe, Madden & Pearce 2009b; Drewe *et al*. 2011). This study found that 75% of the time, the first case of disease observed within a group was a male animal, increasing the evidence for their role in disease introduction. Females tend not to move between groups, rather staying with their natal group, or forming splinter groups with coalitions of males (Doolan & Macdonald 1996; Mares *et al*. 2014). In a previous study, no evidence was found for a link between eviction and disease in female meerkats (Drewe 2010). It seems that the role of males in transferring infection between groups may be attributable to their behavioural movements, rather than them being more disposed to developing disease.

We found age to be an important factor in determining the likelihood of disease. This is not surprising as older animals would have had longer potential exposure time and incubation period (interval between infection and clinical signs). The median latent period (interval between infection and infectiousness) for TB in meerkats has been estimated at just over 12 months (Drewe *et al*. 2011), suggesting animals younger than 1 year are unlikely to show clinical signs of this disease. In the present study, however, 20 deaths were recorded in animals aged less than a year of age. These may be indicative of particularly high infection pressure within these groups, as overall incidence was low in this age group; only 1·3 cases occurred per 100 study years in animals aged less than 1 year. The population at risk declined sharply (see Fig. 2) in early life. Exposure is more likely to occur as these individuals age, and socially interact, than in their early life in the burrow. Age effects are common findings in TB studies of other species, for example in ferrets (*Mustela furo*) (Lugton *et al*. 1997) and badgers (Delahay *et al*. 2013).

Within this hierarchical society, dominance comes at a high cost to the individual (Clutton‐Brock *et al*. 2010), and yet dominance did not affect an animal's chances of being euthanased owing to TB. This lack of effect is perhaps surprising as dominant individuals have the greatest amount of social interaction of all meerkats (Madden *et al*. 2011). However, Drewe (2010) showed that the specific type and direction of interactions must be considered when quantifying disease risk, making the recipients of aggression most likely to become infected. Progression of infection must also be considered; opportunity to become infected is not enough alone to determine disease progression. Social disturbances within the group, particularly those that involve an increase in aggressive interactions such as occur during changeovers in dominant individuals, may be expected to increase the chance of transmission. However, no relationship with disease was observed.

A previous history of TB in the same group predicted the incidence of TB‐related deaths within individuals. Similar effects have been found in cattle and badgers; recent history of TB within a cattle herd is a risk factor for a new breakdown (Skuce, Allen & Mcdowell 2012) and recurrence of TB (Karolemeas *et al*. 2011). In badgers, as here, a greater risk of an individual being infected has been noted when there was a previous history of TB within the social group (Vicente *et al*. 2007; Tomlinson *et al*. 2013a). The frailty term in the model showed that there was important variation between groups, and this is likely to be in part due to TB history within the group. Over half of the groups studied had never had a case of TB. After the index case, further cases were documented for up to 3 years, although it is uncertain as to whether these were related or were the result of reinfection. Were repeat cases found to have been as a result of the index case, then they may be either individuals infected at the same point as the index but slower to develop into cases, or as a result of spread from infected individuals, or via contamination of shared environments such as sleeping burrows.

The incidence of TB‐related deaths was higher between the years 2006–2010 than in the other two time periods covered by our study, and although it is unclear why, environmental factors such as rainfall may play a role. Individual peaks in 2007 and 2012 both came after a year of high rainfall, although no statistical association with rainfall was found. Males more frequently move between groups during times of increased rainfall (Mares *et al*. 2014) and more inter‐group roving might lead to greater transmission of infection. There was weak evidence that low rainfall (below 200 mm) was a risk factor for a group developing disease for the first time, with a hazard ratio at these times of 2·28 (0·91–5·72, *P* = 0·079), and this may be associated with reduced foraging success. A reduction in food availability would likely have implications for immune function. No correlation between body condition score and measured TB infection status was found in a study that examined 258 different meerkats (581 sampling events) from 2005 to 2008 (Drewe 2009). Body condition and weight appear to vary more with age, food availability and pregnancy status.

No group size effect was noted, and having more adults of roving age did not make a group more likely to develop clinical disease. A similar lack of group size effect was noted in badgers (Gallagher 1998). Larger meerkat groups are no more likely to come into contact with other groups and only receive a small increase in the number of roving males visiting compared to smaller groups (Drewe, Madden & Pearce 2009b). The lack of effect is likely to be due to there being little or no increase in contacts with individuals from outside the group. Immigration into the group is a definite example of such contacts, and here an effect was seen. Immigrants will almost always be males (Mares *et al*. 2014), and a history of immigration in the past 12 months increased the hazard by a factor of 3·00 (1·23–7·34, *P* = 0·016). The evidence for an effect of immigrants in the previous 3 or 6 months was weaker and this suggests that a longer latent period is applicable.

As this study focused on clinical cases with a prescriptive case definition, case rates will be an underestimate of the true infection level in the population. Many animals will have been infected without showing clinical signs, or were lost to follow‐up before these were recognised. A study of risk factors for infection would require the implementation of long‐term surveillance. Not only would this identify which animals are initially becoming infected but it would also allow for increased understanding of why some infected animals develop clinical disease, and others appear not to do so. Cases identified in this study are likely to be a good match for the study question, which was to identify those animals with advanced disease. While poor sensitivity is a common feature of TB tests, and the visual case definition used here is likely to have an even lower value than most, the case definition used is highly specific. Given that groups are visited so regularly, the time measurements in the analysis are a reliable basis for the analysis.

In conclusion, our analysis of this long‐term dataset has shown that although disease due to TB does occur in younger animals, it is older animals that are at greater risk. Intra‐group transmission appears to be a major barrier to spread, as has been seen in other species, and once TB is active within a group then the likelihood of others developing the disease rises steeply. Numbers of cases vary between years, and it has been suggested that this may have connections with local rainfall, or availability of susceptible animals. The importance of previous group history for individual risk makes factors influencing a group's TB status, such as immigration, very influential. This study has presented evidence for clustering and found that there are both group‐level and individual‐level risk factors for developing disease. This has implications for control of TB in wild meerkats. These may include targeting vaccinations of groups that experience immigrations, or culling of older animals in diseased groups soon after an initial case. Our findings are similar to those reported in other species such as badgers, and therefore support applicability of these results to aid the management of TB in a broader range of species. Additional data on the infection status of individuals and how this changes over time would have applications to our understanding of transmission and disease progression.

## Authors' contributions

S.P., T.C.B. and J.D. conceived the idea for this work; S.P. designed the methodology, extracted and analysed the data, and led the writing of the manuscript. All authors contributed critically to the drafts and gave final approval for publication.

## Data accessibility

The data used in analysis are available at *figshare*: https://dx.doi.org/10.6084/m9.figshare.4519343.v1 (Patterson 2017).
